# Oral microbiomes of patients with infective endocarditis (IE): a comparative pilot study of IE patients, patients at risk for IE and healthy controls

**DOI:** 10.1080/20002297.2022.2144614

**Published:** 2022-11-15

**Authors:** Jean-Luc C. Mougeot, Micaela Beckman, Bruce J. Paster, Peter B. Lockhart, Farah Bahrani Mougeot

**Affiliations:** a Translational Research Laboratory, Department of Oral Medicine/ Oral Maxillofacial Surgery, Cannon Research Center, Carolinas Medical Center, Atrium Heath, Charlotte, NC, USA; b Department of Microbiology, the Forsyth Institute, Cambridge, MA, USA

**Keywords:** Infective endocarditis, oral microbiome, next-generation sequencing, blood culture, bacterial species

## Abstract

**Background:**

Infective endocarditis (IE) is an uncommon disease with high morbidity and mortality rates, which often develops from oral bacterial species entering circulation.

**Objective:**

We compared oral microbiome profiles of three groups: IE patients (N  9 patients; n = 27 samples), disease controls at risk for IE (N = 28; n = 84), and healthy controls (N = 37; n = 111). Bacterial species in IE patients’ blood cultures were identified for comparison with matched oral samples.

**Design:**

Oral microbiome profiles were obtained from buccal mucosa, saliva, and tongue samples for all three groups and from sub- and supra-gingival plaque samples of the IE group (N = 9; n = 16) and disease controls (N = 28; n = 54). *Alpha*- and *beta*-diversities were determined based on relative abundance data. Discriminative species were identified by LEfSe, *post hoc* Mann-Whitney, and ROC analyses. Identity of the bacterial species in IE patients’ blood cultures was confirmed by *16S-rRNA* gene Sanger sequencing.

**Results:**

*Alpha*- and *beta*-diversities differed between groups. Discriminative IE-associated species were identified, *e.g. Haemophilus parainfluenzae* and *Streptococcus sanguinis*. Two blood isolates were *Staphylococcus aureus*, also identified in one matched saliva sample. *Streptococcus mutans* was present in one patient’s plaque samples and blood culture.

**Conclusions:**

Oral microbiomes of IE, non-IE disease controls, and healthy controls differed significantly. A better understanding of IE-related bacterial-host interactions is warranted.

## Introduction

Infective endocarditis (IE) is an uncommon but serious disease, with a high morbidity and mortality rate with an average hospital cost of $120,000 per patient [1,2]. IE involves infection of the heart endothelial tissues, typically heart valves. Diagnosis of IE, per the Modified Duke criteria, requires both pathological and clinical evidence [3]. The American Heart Association (AHA) defines people as being at high risk (*e.g*. people with prosthetic heart valves), moderate risk (*e.g*. people with heart murmurs such as mitral valve prolapse), or at low risk.

IE can be acquired by a variety of invasive procedures that introduce bacteria into the blood stream (e.g. dialysis, injection drug use, surgical procedures) and in most cases requires a defective heart valve for platelet formation as a growth medium for bacteria [1]. Bacteria may come from the oral cavity, gut, or skin [2,4,5]. Previous studies by our group have focused on the incidence, nature, duration, and magnitude of bacteremia originating from the oral cavity [6,7,8]. We detected putative IE bacterial pathogens in blood of volunteers following tooth extraction or tooth brushing and demonstrated an association between these surrogate markers for risk of IE with the oral health status of the study participants [9].

The role of oral hygiene and resultant oral disease have long been implicated in the development of IE through bacteremia [10–17]. A study by Beutler et al. reported that bacteremia was detected in 30–40% of patients receiving initial or supportive periodontal therapy [18]. Another study by Horliana et al. showed bacteremia following periodontal procedures in about 50% of patients [19]. Furthermore, a recent study by Emery et al., investigating the microbiome of blood in populations of periodontal health and disease, found 78 of 150 identified species to be of oral origin as defined by the Human Oral Microbiome Database [20]. About 20–54% of IE cases are allocated to oral species, although some of these typically may colonize the skin, gut or other anatomical areas [21–24].

The purpose of this study was to conduct a species-level analysis of the oral microbiome in patients with AHA-defined ‘moderate risk’ for IE who developed IE, with those who did not, and with healthy controls. Approximately 90% of people at risk for IE are in this AHA moderate risk group [25–27]. Although historically the concern has been focused on invasive dental procedures, routine daily activities such as tooth brushing can also cause a bacteremia leading to IE [6,9,28]. In this study, we used next-generation sequencing (NGS) of the v1-v3 hypervariable region of the *16S rRNA* gene to identify over 700 known oral bacterial species [29–32,33,34,35,36]. Furthermore, we analyzed whether there was a significant link between the species found in blood and in the oral cavity of IE patients.

## Methods

### Study population

Study participants were recruited at Carolinas Medical Center–Atrium Health, Charlotte, NC. The study was approved by the Institutional Review Board (IRB # 00019606) and participants signed an informed written consent. The study cohort (N = 74) was stratified into healthy controls (HC; N = 37, three oral sample sites [n = 111]), non-IE outpatients at moderate risk for IE (DC; N = 28, five oral sample sites [n = 138]), and recently hospitalized patients with IE, who had also been at moderate risk for IE (IE; N = 9, five oral sample sites [n = 43]). Moderate risk for IE was defined based on AHA criteria [37]. Patients with AHA-defined ‘high risk’ and those with healthcare associated, or injection drug-related IE were excluded from the study. IE patients were recruited for the study within 24 h of admission to the hospital.

### Clinical oral examination

Subjects were examined by a dentist or dental hygienist for assessment of their oral health status. Six Ramfjord index teeth were examined, and an adjacent tooth was used if an index tooth was missing. The primary outcome was dental calculus index, and secondary outcomes were the plaque index, gingival index, and periodontal pocket depth, bleeding on probing, and clinical attachment level measurements.

### Sample collection and processing

Oral samples were collected prior to the clinical oral examination and no less than 1 h from eating, drinking or personal oral care. Subjects were asked to rinse their mouth briefly with water five min prior to sample collection. Oral samples were collected in the following order:

#### Saliva

A fresh, unstimulated 1 mL sample of saliva (S) was collected for 2 to 5 min in OMNI-501 tubes (OmniGene-Discover OMNI-501, DNA Genotek Inc., Ottawa, ON, Canada), following kit instructions. Samples were processed according to the manufacturers’ instructions (DNA Genotek Inc.) and stored at −80°C.

#### Oral mucosal bacterial swabs

Buccal mucosa (B) samples were obtained by swabbing both sides of the buccal mucosa for 10s each. Tongue (T) samples were obtained by swabbing

an approximately 1 cm^2^ region on both sides of the mid-dorsal surface of the tongue for 5 s.

Swabs were suspended in PBS with 0.04% sodium azide and rotated using an orbital shaker for at least 2 h at room temperature to release bacteria from the swabs into the solution. The sample suspensions were centrifuged, and cell pellets were stored at −80°C.

Dental plaque samples: Following assessment of all clinical periodontal measures, supragingival [supra-g] and subgingival [sub-g] samples were obtained separately for each index tooth, with a single swipe using individual sterile Gracey curettes [38,39]. If an index tooth was missing, the plaque samples were collected from an adjacent tooth. Samples were dislodged from the curette and resuspended in 250 µl nuclease-free PBS. The suspensions were centrifuged, and pellets were stored at −80°C.

#### Bacterial DNA extraction and next generation sequencing

Bacterial genomic DNA was extracted from frozen pellets using the ZymoBIOMICS Targeted Metagenomic Sequencing −96 MagBead DNA kit (Zymo Research, Irvine, CA). Quick-16S Primer Set v1-v3 (Zymo Research, Irvine, CA) was used to prepare the sequencing library using real-time PCR to control cycle number and limit PCR chimera formation. Final PCR products from all samples were quantified with qPCR fluorescence readings and pooled based on equal molarity. Pooled PCR library was cleaned up with the Select-a-Size DNA Clean & Concentrator (Zymo Research, Irvine, CA). Final quantification was performed with TapeStation (Agilent Technologies, Santa Clara, CA) and Qubit (Thermo Fisher Scientific, Waltham, MA). The library was sequenced on Illumina MiSeq with a v3 reagent kit of 600 cycles. The Dada2 pipeline was used to identify amplicon sequences from raw reads [40]. Uclust from Qiimev1.9.1 was used for taxonomy assignment with the Zymo Research Database (16S database) [41, Zymo Research, Irvine, CA].

#### Blood cultures and bacterial identification

Blood samples from IE patients (N = 9) were collected, cultured, and bacterial isolates identification was performed in our clinical microbiology laboratory (Carolinas Medical Center–Atrium Health) as part of standard clinical care. Identity of the blood culture isolates was also verified by *16S-rRNA* gene sequencing. DNA was isolated from the cultured isolates using the QIAamp DNA Mini Kit (Qiagen GmbH, Hilden Germany) according to the manufacturer’s instructions and subjected to *16S-rRNA* gene Sanger sequencing (Psomagen, Inc., Rockville, MD) as described before [7,42]. Sequences from FASTA files were processed using the top match in BLASTn [43]. The nine FASTA files were then merged using python3 Biopython library with identity matches from BLASTn combined with the sample name to form the ID of each FASTA [44].

### Bioinformatics analysis

#### Alpha-diversity

Shannon and Simpson indices were generated using PRIMER_v7_ software (PRIMER-E Ltd., Ivybridge, UK) from relative abundance data for BST sample site data combinations of HC *vs*. IE, HC *vs*. DC, and DC *vs*. IE as well as for sub-g and supra-g sample site data combination and sub-g alone of DC *vs*. IE. Wilcoxon rank sum test was used to determine significance (α = 0.05). Boxplots were generated using Primer_v7_.

#### Beta-diversity

PRIMER_v7_ was used to perform cross-sectional comparisons. Relative abundance (RA) data were square root-transformed and converted to Bray-Curtis similarity matrices followed by PERMANOVA analyses using mixed-model with unrestricted permutation of raw data, 9,999 permutations, and type III partial sum of squares as previously implemented by our group [32]. Fixed factors in the PERMANOVA design were ‘Diagnosis’ (disease status HC, DC, and/or IE) and ‘Site’ (B, S, T, sub-g, and/or supra-g). Monte-Carlo corrected p-values (α = 0.05) were determined. Comparisons included BST sample site data combinations for HC *vs*. DC, HC *vs*. IE, and DC *vs*. IE. Additional sub-analyses were completed for the DC *vs*. IE comparison using the sub-g and supra-g sample site data combination and the sub-g sample site data alone in the PERMANOVA design.

#### Linear discriminant analysis (LDA) effect size (LEfSe)

Tabular text files were formatted to perform LEfSe analysis using the online tool Galaxy_v1.0_ and normalized [45]. Comparisons included the BST sample site data combinations for HC *vs*. DC, HC *vs*. IE, and DC *vs*. IE. LEfSe input consisted of ‘Diagnosis’ (disease status HC, DC, and/or IE) as LEfSe ‘Class’ option and ‘Subject’ as LEfSe ‘Subject’ option. Using the all-against-all strategy for multi-class analysis [46] the factorial Kruskal-Wallis and pairwise Wilcoxon signed rank test were set at a Monte-Carlo corrected significance of α = 0.05 to calculate LDA scores. The log LDA score was set at a threshold of > 4.0 for all comparisons other than HC vs. DC which was set at a threshold of > 2.0. Histograms of the significant biomarkers were plotted at the genus level and taxa at the species level were investigated for their role in IE using PubMed (https://pubmed.ncbi.nlm.nih.gov).

#### ROC analysis

Taxa at the species level identified by LEfSe, and Mann-Whitney U-test significant (p < 0.05), were subjected to Receiver Operating Characteristic (ROC) analysis using relative abundance (RA) data with MedCalc_v19.2.6_ software (MedCalc Software Ltd, Ostend, Belgium; https://www.medcalc.org; 2020). ROC curves and integrative dot plots were created for DC vs. IE, HC vs. DC, and DC vs. IE BST as well as DC vs. IE using sub-g and supra-g, and sub-g sample data.

#### MIND network analysis

The Microbial Interaction Network Database (MIND) online tool was used to determine interactions of species for the BST sub-g and supra-g sample site data combination to determine overlaps between the LEfSe identified differential DC or IE species compared to BLASTn identified blood isolate species.

## Results

**Figure 1.**
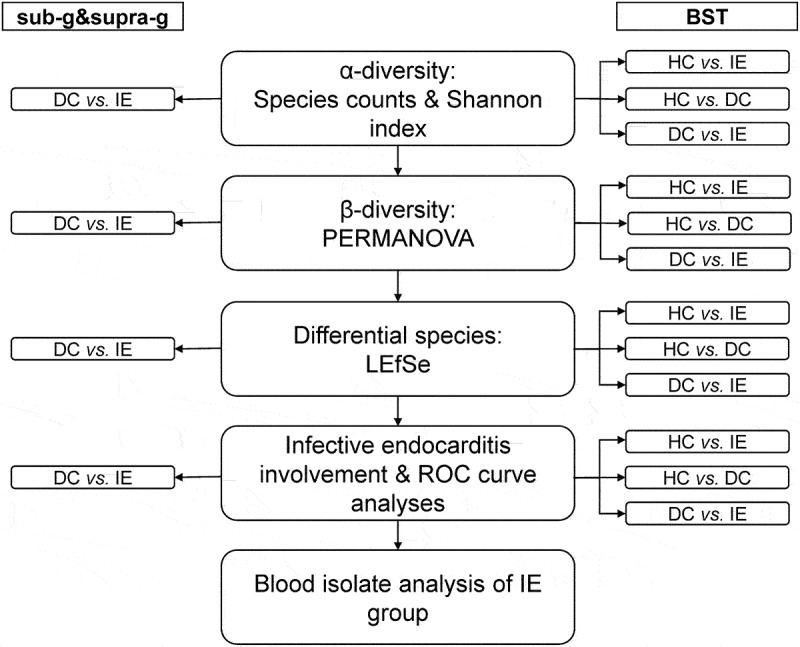
Analytical design.

The overall analytical strategy for oral microbiome community analysis is presented in Figure 1. Demographics and dental clinical characteristics between the three groups were overall balanced regarding age and gender distribution, based on Wilcoxon rank sum and chi-squared tests (α = 0.05), except for an age difference between HC and DC groups (Table 1). There were no significant differences regarding the average calculus or plaque index scores between the DC and IE groups.

**Table 1.** Subjects’ demographics and dental clinical characteristics for patients at moderate risk for IE who had IE or did not, and healthy controls.

Criteria	HC^a^	DC^b^	IE^c^	HC *vs*. DC	HC *vs*. IE	DC *vs*. IE
		data			p-values^d^	
Subject Count (M/F)	37 (11/26)	28 (14/14)	9 (5/4)	0.1597 USD	0.2852 USD	1.00 USD
**Age:**				0.007*	0.250*	0.366*
Mean	52.6	62.3	58.4			
Median	53	65	57			
SD	14.4	15.3	13.2			
Range	24–84	25–87	33–77			
**Ethnicity Count:**						
M: C/AA/O	7/0/4	10/4/0	5/0/0	0.0975*	0.1814*	0.1814*
F: C/AA/O	21/2/3	8/6/0	4/0/0	0.0591*	0.1003*	0.1814*
**Whole mouth average PD (SD)**	NC	2.25 (± 0.58)	2.36 (±0.76)			0.449*
**Whole mouth average CI (SD)**	NC	0.34 (± 0.62)	0.63 (± 0.81)			0.163*
**Oral sample combinations:**						
**BST**	111	84	27			
**sub-g&supra-g**	NC	54	16			
**sub-g**	NC	28	8			

**^a^**Healthy control (HC) group

**^b^**Non-infective endocarditis (IE) disease control (DC) group consisting of individuals at moderate risk for IE per AHA criteria, who did not have IE

**^c^**Individuals at moderate risk for IE who developed IE

**^d^**p-values of group comparisons were obtained using either Chi-squared test or Wilcoxon rank sum test

M is Male; F is female; C is Caucasian; AA is African American; O is other; PD is pocket depth; CI is calculus index; SD is standard deviation; BST is buccal mucosa, saliva, and tongue; sub-g&supra-g is subgingival and supragingival plaque; sub-g is subgingival plaque alone; NC is not collected.

Statistical analyses were completed in R:

^$^Chi-squared test p-value

*Wilcoxon rank sum test p-value

### Alpha- and beta-diversities

**Figure 2.**
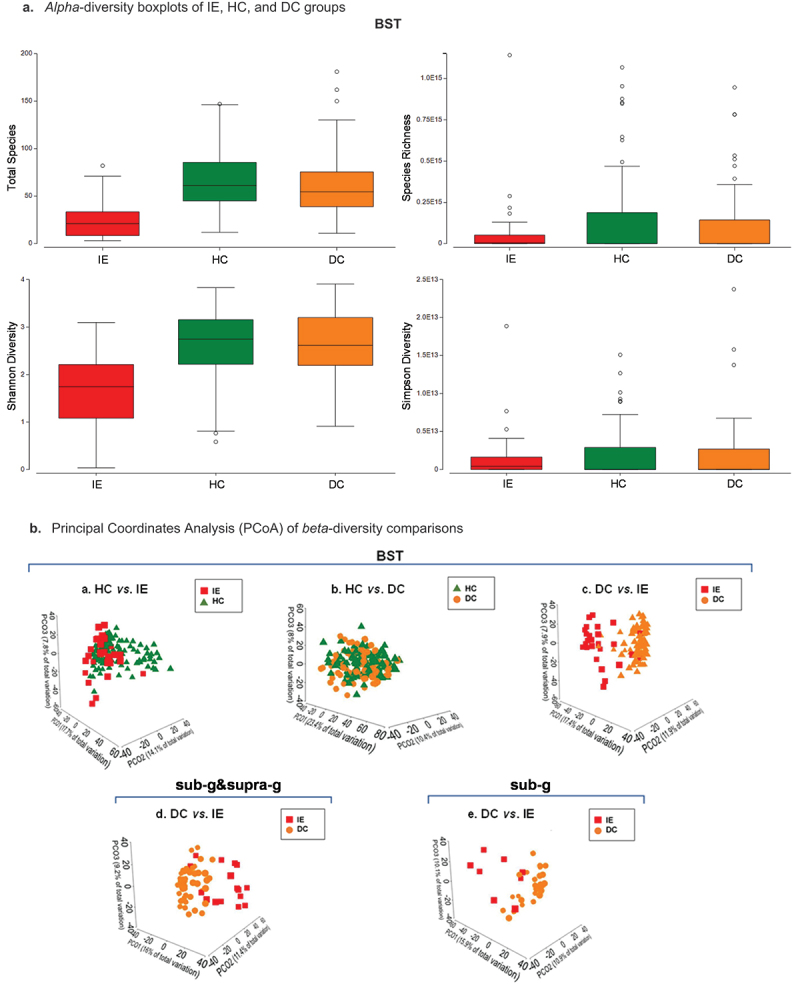
*Alpha*- and *beta*-diversity results. *Alpha*-diversity boxplots of IE, HC, and DC groups. Principal Coordinates Analysis (PCoA) of *beta*-diversity comparisons. a. Boxplots showing the total species (top left), species richness (top right), Shannon diversity indices (bottom left), and Simpson diversity indices (bottom right) of oral samples taken from the buccal mucosa (B), saliva (S), and tongue (T) (BST sample data combination) for patients diagnosed with infective endocarditis (IE; red), non-IE disease controls (DC; orange), and healthy controls (HC; green). b. Principal coordinates analysis (PCoA) of relative abundance data for sample site data combinations of buccal mucosa (B), saliva (S), and tongue (T) combination for a. healthy controls (HC) (green triangles) *vs*. infective endocarditis (IE) patients (red squares), b. HC *vs*. non-IE disease controls (DC) (orange circles), and c. DC *vs*. IE patients. Other comparisons included d. subgingival plaque (sub-g) data combined with supragingival plaque (supra-g) or e. sub-g data alone for DC *vs*. IE patients.

*Alpha*-diversity results are presented in Table 2. All BST sample data combination comparisons except for HC *vs*. DC were significant (p < 0.05) (Figure 2a). The combined sub- and supra-gingival plaque data and subgingival plaque alone comparisons of DC *vs*. IE were found to be significant with p-values < 0.01. All *beta*-diversity comparisons were found to be significant (Monte-Carlo corrected p < 0.001) for disease status HC, DC, and IE groups (Table 3). Principal coordinates analysis (PCoA) plots of each comparison are presented in Figure 2b. The IE group was found distinct from the HC and DC groups, whereas the DC group was not found different from the HC group based on overall relative abundance data.

**Table 2.** *Alpha-*diversity analyses show significant differences for HC *vs*. IE and DC *vs*. IE comparisons.

Comparison^a^	Min^b^	Max^c^	Avg^d^	Stdev^e^	p-value^f^
**HC vs DC BST**					
HC	12	147	66.5	27.3	0.819
DC	11	181	60.6	32.1	
**HC vs IE BST**					
HC	12	147	66.5	27.3	5.73x10^−07^
IE	3	82	25.4	20.2	
**DC vs IE BST**					
DC	11	181	60.6	32.1	1.76x10^−06^
IE	3	82	25.4	20.2	
**DC vs IE sub-g &supra-g**					
DC	22	186	81.9	34.4	4.22x10^−05^
IE	13	133	44	33.6	
**DC vs IE sub-g**					
DC	26	186	87.9	34.3	0.003
IE	22	100	47.5	29.9	

**^a^**Comparisons consisted of healthy controls (HC), disease controls (DC), and/or infective endocarditis (IE) patient groups with sample data combinations of oral swabs of buccal mucosa (B) and tongue (T) with saliva (S) or data combinations of supra- and subgingival plaque (supra-g&sub-g) or subgingival plaque (sub-g) alone

**^b^**Minimum number of species detected per sample

**^c^**Maximum number of species detected per sample

**^d^**Average number of species detected per sample

**^e^**Standard deviation of species detected per sample

**^f^**Wilcoxon rank sum test significance (α = 0.05)

**Table 3.** PERMANOVA *p*-values for healthy controls, non-IE disease controls, and IE patient comparisons show significant *beta*-diversity changes.

Comparison^a^	Disease status p-value^b^	Site p-value^c^
**HC vs DC^d^**		
BST	0.0008	0.0001
**HC vs IE^e^**		
BST	0.0001	0.0001
**DC vs IE^f^**		
BST	0.0001	0.0001
sub-g&supra-g	0.0001	0.5421
sub-g	0.0002	NA

**^a^**Comparisons of healthy controls (HC), non-IE disease controls (DC), and infective endocarditis (IE) patients

**^b^**Disease status Monte-Carlo corrected p-value

**^c^**Site Monte-Carlo corrected p-value

Sample site data combinations included buccal (B), saliva (S), tongue (T), subgingival plaque (sub-g), and/or supragingival plaque (supra-g). PERMANOVA’s were completed using relative abundance square root transformed data and a resemblance S17 Bray-Curtis similarity matrix. PERMANOVA’s were run using type III partial sum of squares, permutation of residuals under a reduced model, with 9,999 permutations, and a Monte-Carlo corrected significance p-value < 0.05. NA: not applicable

### LEfSe

LEfSe analysis was able to differentiate seven HC taxa and 17 DC taxa from the HC vs. DC BST sample site data combination comparison. When comparing BST samples from HC vs. IE, LEfSe identified eight and six distinctive taxa for HC and IE, respectively. Furthermore, the BST combination of DC vs. IE returned six differential features for DC and five differential features for IE. Sub-analyses of DC vs. IE revealed four and six DC and IE distinctive taxa, respectively, for the sub- and supra-gingival plaque sample data combination. Additionally, analysis on DC vs. IE sub-gingival plaque alone identified five DC taxa and one IE taxon. Histograms showing species identified as differential features for all comparisons and the Wilcoxon rank sum test significance are shown in Figure 3. A table of LEfSe results and species involved in bacteremia and/or IE using PubMed are presented in Supplemental Table 1.

**Figure 3.**
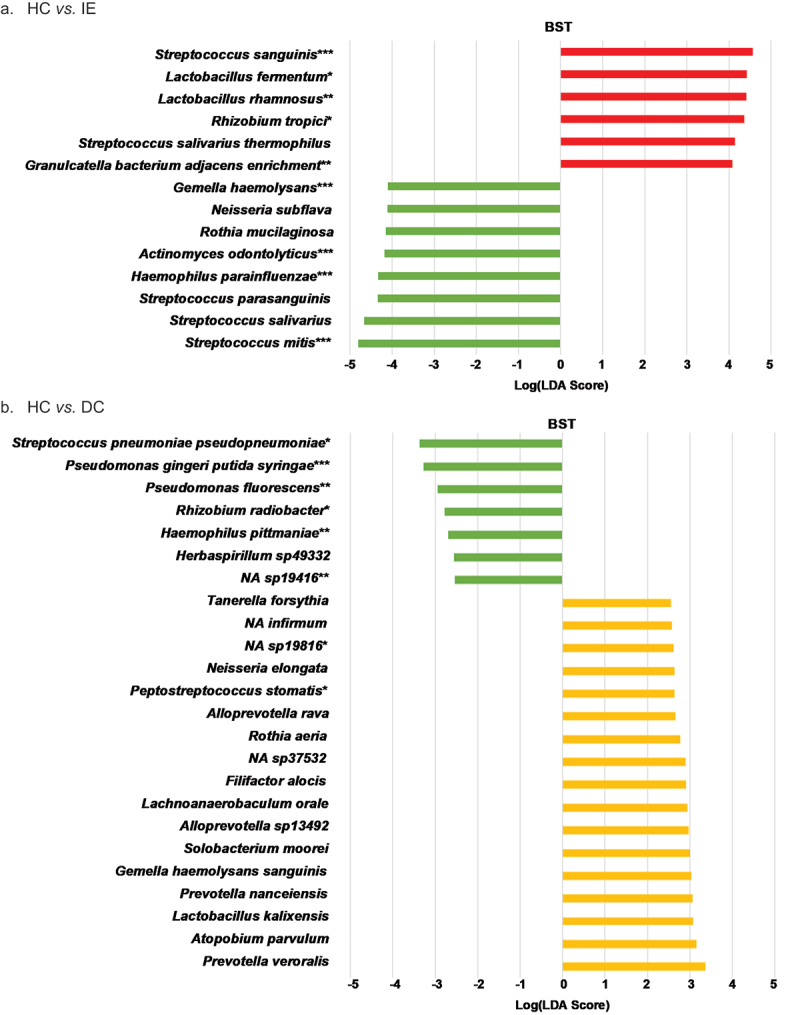
LEfSe histograms comparing HC, IE, and DC groups. HC *vs*. IE. HC *vs*. DC. DC *vs*. IE. Linear discriminant analysis (LDA) effect size (LEfSe) of healthy controls (HC), non-IE disease controls (DC), and infective endocarditis patients (IE) based on relative abundance data. Comparisons HC *vs*. IE (**a**), HC *vs*. DC (**b**) and DC *vs*. IE (**c**) were performed. Samples sites included buccal mucosa (B), saliva (S), tongue (T), subgingival plague (sub-g), and/or supragingival plaque (supra-g). LEfSe input consisted of ‘Diagnosis’ (disease status HC, DC, and/or IE) as LEfSe ‘Class’ option and ‘Subject’ as LEfSe ‘Subject’ option. Using the all-against-all strategy, the factorial Kruskal-Wallis and pairwise Wilcoxon signed rank test were set at a Monte-Carlo significance of α = 0.05 to calculate LDA scores. The log LDA was set at a threshold of > 4.0 for all comparisons other than HC *vs*. DC which was set at a threshold > 2.0. An asterisk (*) denotes significance of Mann-Whitney U-test. *< 0.05; **< 0.01; ***< 0.001.

**Figure 3.**
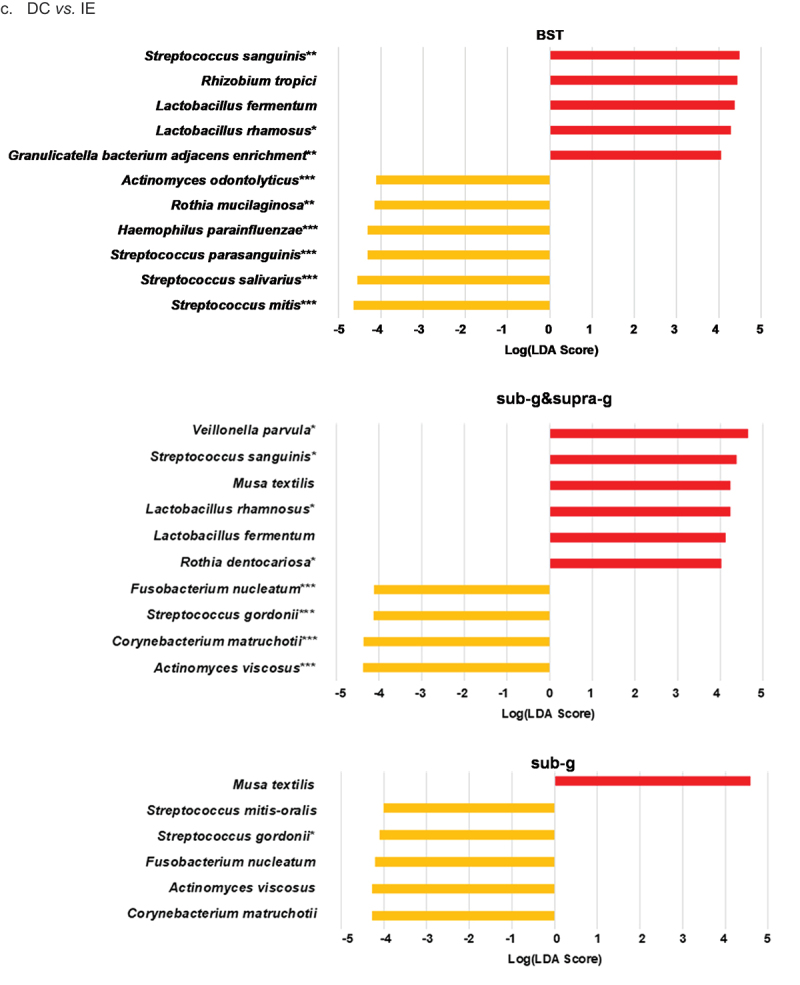
(Continued.)

### ROC analysis

**Figure 4.**
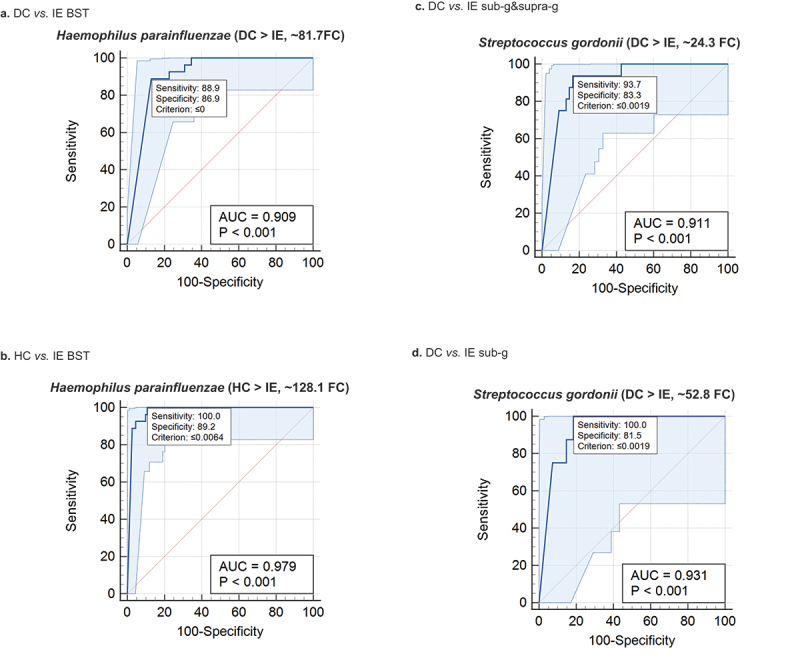
Receiver Operating Characteristic (ROC) curve analysis. DC *vs*. IE BST. HC *vs*. IE BST. DC *vs*. IE sub-g&supra-g. DC *vs*. IE sub-g.Receiver Operating Characteristic (ROC) curves based on relative abundance data were determined for each comparison of healthy controls (HC), non-IE disease controls (DC), and/or infective endocarditis patients (IE) groups for the sample data combinations of buccal mucosa (B), saliva (S), tongue (T), subgingival plaque (sub-g) and/or supragingival plaque (supra-g). No species were found to have an area under the curve > 0.6 for the HC *vs*. DC BST comparison. Mean relative abundance changes of each species probe in the form of fold changes (FC) are shown for each group. The (>) sign is used to depict the group with highest abundance on average. The criterion is the cut-off point selected to discriminate between two populations.

No species were found to have AUCs > 0.6 for the HC *vs*. DC BST comparison. Regarding the sample data combination BST for HC vs. IE, we identified one ROC with an AUC > 0.9, namely, *Haemophilus parainfluenzae*. The ROC of three species (*Gemella haemolysans, Actinomyces odontolyticus*, and *Streptococcus mitis*) from the HC vs. IE comparison had AUCs between 0.8 and 0.9. These species were more abundant on average in the HC compared to the IE group. *H. parainfluenzae* also had an AUC > 0.9 in the DC vs. IE BST comparison but there were also four species more abundant on average in the DC group with ROCs with AUCs between 0.8 and 0.9 (*A. odontolyticus, Streptococcus parasanguinis, Streptococcus salivarius*, and *S. mitis*). *Streptococcus gordonii* had an AUC of 0.911 for the sub-analysis of DC *vs*. IE (sub-g and supra-g) which was also found to be the most distinctive species in the sub-g alone comparison. ROC plots depicting *H. parainfluenza*e and *S. gordonii* (overrepresented in DC and HC vs. IE group) are shown in Figure 4. An ROC analysis summary is shown in Supplemental Table 2.

### Blood isolate analysis

The FASTA files corresponded to nine taxa determined by BLASTn with ≥ 96% identity. Two blood isolate cultures were identified as *Staphylococcus aureus*. The other seven unique cultures were *Staphylococcus saprophyticus, Enterococcus faecalis, Streptococcus oralis, Staphylococcus agalactiae, Streptococcus mutans, Cardiobacterium hominis*, and *Serratia marcescens* (Supplemental Figure 1).

### MIND analysis

The BST sample site data combination analysis of the LEfSe results of the species characterizing the DC group in the DC *vs*. IE comparison (Figure 3c) resulted in an output of 151 interactions (input = six species present in the MIND database including all six LEfSe identified species). These interactions included 59 overlapping with those found using the BLASTn identified blood isolate species using as an input seven MIND species out of eight blood isolate species, which resulted in an output of 115 interactions (Supplemental Table 3a, Supplemental Figure 1). The analysis of the BST IE group from the LEfSe DC *vs*. IE comparison, based on an input of four MIND species out of five LEfSe species, yielded an output of 27 interactions including only seven overlapping species interactions (Figure 3c; Supplemental Table 3a). When comparing the blood isolates to the sub-g and supra-g sample site data combination for the DC group (input = three MIND of four LEfSE species; output = 55 interactions) and IE group (input = five MIND out of six species; output = 73 interactions), we were able to identify 38 and 27 overlapping interactions, respectively (Supplemental Table 3b). A MIND network of blood isolate species is shown in Supplemental Figure 2.

## Discussion

This is the first study to characterize the microbiome of the oral cavity using saliva, tongue, buccal mucosa, supra- and subgingival plaque samples of IE patients, people at risk for IE *vs*. healthy controls. Using *16S-rRNA* gene v1-v3 Illumina sequencing, we were able to identify 711 species across all sample sites. Additionally, we were able to show that species diversity is lost in patients with IE when compared to either healthy or non-IE disease controls (Figure 2a). Our data suggest that IE is associated with *beta*-diversity changes when comparing each group (IE *vs*. HC, HC *vs*. DC, and DC *vs*. IE) (Figure 2b).

It has been well established that oral microbiome communities differ by site [47–49]. When comparing sites (B, S, T, sub-g and supra-g) for the IE and DC groups, we found most DC species taxa present at each site had higher mean relative abundance than IE species. In saliva, 102 taxa out of 711 identified had higher mean RA in the IE group than the DC group. Furthermore, at B, T, sub-g and supra-g sites IE only had higher relative abundance in 70, 90, 101, and 87 species taxa, respectively. By comparing HC *vs*. IE and HC *vs*. DC (B, S, and T sites), we found similar results. However, the HC *vs*. DC comparison had more species taxa where the relative abundance was higher at each site in DC than HC (B = 231 species taxa; S = 235 species taxa; T = 270 species taxa).

Using LEfSe analysis, we were able to distinguish significant features of each group. Surprisingly, we found species normally associated with IE to be differential features of the healthy and non-IE disease control groups. In buccal/ saliva/ tongue sample data combination comparisons of DC *vs*. IE and HC *vs*. IE, *S. mitis*, a *viridans* group species dominant in the oral cavity and pharynx, was the most differential feature for the HC and DC groups. Recently, *S. mitis* was identified as a cause for IE in a patient with a ventricular septal defect undergoing an orthodontic procedure [50].

Furthermore, *Streptococcus sanguinis* was the most differential feature for IE in both comparisons and was identified in the blood culture of a patient presenting with IE (Figure 3(a, c)) [51]. In the HC *vs*. DC (BST) comparison, *Streptococcus pneumoniae -pseudopneumoniae* was the most differential for HC while *Prevotella veroralis* was the most differential for DC (Figure 3b). A systematic review by de Egea et al. concluded that *S. pneumoniae* are responsible for up to 3% of IE cases in the post-antibiotic era [52]. In the sub-g and supra-g sample site data combination of DC *vs*. IE, *Actinomyces viscosus* and *Veillonella parvula* were the most differential features for DC and IE, respectively (Figure 3d). *V. parvula* was reported as a cause for bacteremia in an 82-year-old patient with no recent history for dental procedures, infections or surgeries [53]. For the sub-g only comparison, *Corynebacterium matruchotii* and plant mitochondria/chloroplast from *Musa textilis* were the most differential features for DC and IE, respectively [54] (Figure 3e).

ROC analysis showed that in BST samples, *H. parainfluenzae* was underrepresented and the most distinctive species in the IE group compared to the DC group (AUC = 0.91). It is well established that *Haemophilus* spp. are grouped as HACEK organisms inhabiting the mouth and upper respiratory tract and are capable of causing IE [55]. Also, in supra- and subgingival plaque samples *S. gordonii*, overrepresented in the DC group *vs*. the IE group, was the most distinctive species (AUC > 0.90) (Supplemental Table 1). While *Streptococcus cristatus, S. gordonii, S. mitis, S. parasanguinis*, and *S. salivarius* had, on average, a higher relative abundance in the DC group compared to the IE group for all oral site combinations analyzed, the opposite was true for *S. sanguinis*. We were also able to identify *S. mutans* in supra- and subgingival plaque samples and the corresponding blood sample in an IE patient. In a study by Carinci et al., they concluded that 90% of IE cases are caused by *Streptococcus, Staphylococcus*, or *Enterococcus* species. Our data support these results, as more than 77% of blood culture isolates belonged to these genera (Supplemental Figure 1).

Additionally, in our study, blood isolate analysis correlated *S. aureus* as a blood isolate of one patient with its matched saliva sample. Staphylococcal infections are known to pose a challenge in the treatment of IE, likely due to their ability to display methicillin resistance allowing it to evade *beta*-lactam antibiotics such as penicillin, cephalosporin, and carbapenem. Furthermore, staphylococcal infections can cause aggressive destruction of the cardiac valves, compared to much less aggressive disease from streptococci [56,57]. A study characterizing outcomes of patients with IE reported that staphylococcal infections were associated with an increased risk of in-hospital death [58]. Additionally, a study by Pant et al. reported that *S. aureus* accounted for 40% IE cases in the US in 2011 [59]. *S. aureus* has been established as an opportunistic pathogen that lives on the skin and in the nares of approximately 30% of people, but more recently it has been suggested as a common species of the oral cavity [57,60,61]. Other bacterial species known for their involvement in IE include the taxa *Haemophilus* spp., *Aggregatibacter actinomycetemcomitans, C. hominis, Eikenella corrodens*, and *Kingella kingae* constituting the acronymized ‘HACEK’ group [62]. In our IE patient cohort, only *C. hominis* was identified (Supplemental Figure 1).

### Limitations of the study

The number of IE patients was small due to recruitment challenges inherent to the patient population. Nevertheless, we acquired an adequate number of samples from different oral sites to conduct the study for statistical considerations. Indeed, notable differences in *alpha* and *beta*-diversity could be identified when the IE group was compared to healthy controls and the non-IE disease control group. Clinical information about prophylactic antibiotic use was not available for our study cohort. Therefore, we cannot address the effects this may have had on the relative abundance of susceptible species. However, IE patients were recruited for this study as early in their hospitalization as possible (mean time of 24 h) to minimize the impact of interventions such as systemic antibiotics treatment. According to numerous prior studies [63–65], including our studies (Mougeot et al., 2020a; Mougeot et al., 2020b), there may not be large effects on the overall oral microbiome composition of samples taken within a short time following antibiotic intake. Accordingly, while the relative abundance of certain species may have been affected, we did not observe excessive scattering of IE samples in our PCoA analysis that would be characteristic of antibiotic effects.

## Conclusions

Significant oral microbiome differences were observed between IE and non-IE patients at risk for IE (DC group) or healthy controls. Except for *S. sanguinis, Streptococcus* species that had been previously associated with IE were more abundant in the non-IE risk group than in the IE group. There was little correspondence between IE species present in the oral cavity and those present in blood when considering a single IE individual. There is a need for systematic sampling in patients at risk for IE, since IE-associated oral bacteria might colonize heart valves from an oral microbiome reservoir fluctuating or diverging over time. In addition, further studies are needed to understand bacterial networks and bacterial-host interactions associated with IE etiology [66,67].

## Supplementary Material

Supplemental Material

## Data Availability

All data are available *via* the Translational Research Lab Github repository (https://github.com/mbeckm01/IE_Project)
