# Supplementary material for: Oral microbiomes of patients with infective endocarditis (IE): a comparative pilot study of IE patients, patients at risk for IE and healthy controls
Source: J Oral Microbiol. 2022 Nov 15;15(1):2144614. doi: 10.1080/20002297.2022.2144614 (PMC9668282; doi:10.1080/20002297.2022.2144614)
Supplement: Supplemental Material [file ZJOM_A_2144614_SM4896.zip › Supplementary files/Suppl Fig1 IE blood isolate phylogram 10 20 2022 Final.pdf]

Supplemental Figure 1. IE patients' blood isolate phylogram

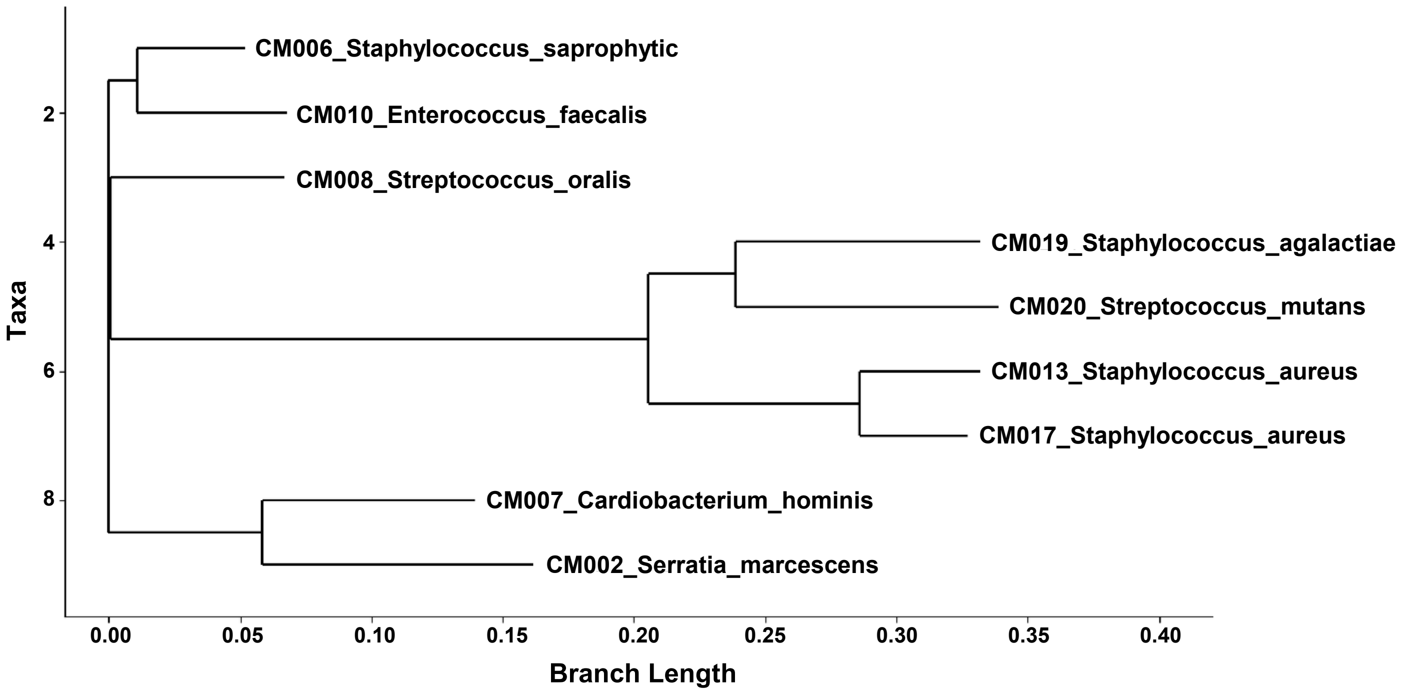

Legend. Phylogram of IE patients' blood isolates (n=9) is shown. A total of 9 taxa identified with >96% identity is represented. Labels show sample name with returned match from BLASTn. Alignments of FASTA files were completed using MUSCLE. Phylogram and calculated distances were created using python3 Biopython package.
