# Supplementary material for: Oral microbiomes of patients with infective endocarditis (IE): a comparative pilot study of IE patients, patients at risk for IE and healthy controls
Source: J Oral Microbiol. 2022 Nov 15;15(1):2144614. doi: 10.1080/20002297.2022.2144614 (PMC9668282; doi:10.1080/20002297.2022.2144614)
Supplement: Supplemental Material [file ZJOM_A_2144614_SM4896.zip › Supplementary files/Suppl Fig2 IE Blood Isolate MIND 10 20 2022 Final.pdf]

Supplemental Figure 2. Blood isolates MIND analysis

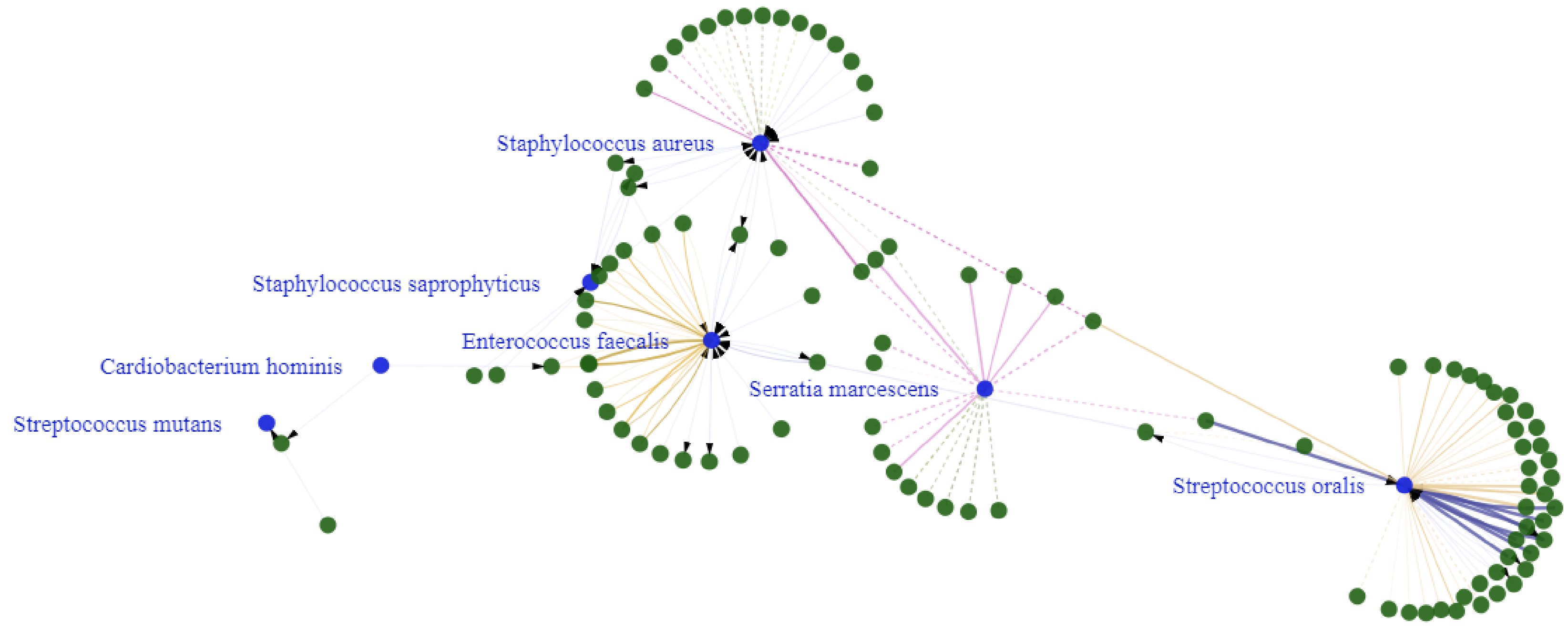

Legend.

The Microbial Interaction Network Database (MIND) online tool was used with an input of Basic Local Alignment Search Tool for nucleotides (BLASTn) identification of blood isolates (blue) from IE patients to view microbial interactions at the species level (green).
