# Supplementary material for: Oral microbiomes of patients with infective endocarditis (IE): a comparative pilot study of IE patients, patients at risk for IE and healthy controls
Source: J Oral Microbiol. 2022 Nov 15;15(1):2144614. doi: 10.1080/20002297.2022.2144614 (PMC9668282; doi:10.1080/20002297.2022.2144614)
Supplement: Supplemental Material [file ZJOM_A_2144614_SM4896.zip › Supplementary files/Suppl Table 1 LEfSe IE and bacteremia 10 20 2022 Final.pdf]

Supplemental Table 1. Species found significant by LEfSe analysis in our study that have been previously involved in bacteremia or infective endocarditis

Comparison[a]	# of species[b]	Species identified in bacteremia/IE[c]	PMID/s[d]
**HC *vs.* IE BST**			
HC	8	*Haemophilus spp., Streptococcus salivarius, Streptococcus mitis*	35986339; 34522379; 35236702
IE	6	*Streptococcus sanguinis*	35976241
**HC *vs.* DC BST**			
HC	7	*Haemophilus* spp.; *Streptococcus pneumoniae-pseudopneumoniae*	35986339; 26426629
DC	17		
**DC *vs.* IE BST**			
DC	6	*Haemophilus* spp.*, Streptococcus salivarius, Streptococcus mitis*	35986339; 34522379; 35236702
IE	5	*Streptococcus sanguinis*	35976241
**DC *vs.* IE sub-g &supra-g**			
DC	4	*Actinomyces viscosus*	34684101
IE	6	*Streptococcus sanguinis; Veillonella parvula*	35976241; 35440093
**DC *vs.* IE sub-g**			
DC	5	*Streptococcus mitis-oralis*	35236702; 36114681
IE	1		

Footnote:

[a]Oral sample site data combinations from samples of buccal mucosa (B), saliva (S), tongue (T), supragingival (supra-g), and/or subgingival (sub-g) plaque for patients who developed infective endocarditis (IE), non-IE disease controls (DC), and healthy controls (HC).

[b]The number of species identified as significant by linear discriminant analysis effect size (LEfSe).

[c]Species identified in previous publications of patients presenting with bacteremia or IE.

[d]PubMed identification number.
