# Supplementary material for: Oral microbiomes of patients with infective endocarditis (IE): a comparative pilot study of IE patients, patients at risk for IE and healthy controls
Source: J Oral Microbiol. 2022 Nov 15;15(1):2144614. doi: 10.1080/20002297.2022.2144614 (PMC9668282; doi:10.1080/20002297.2022.2144614)
Supplement: Supplemental Material [file ZJOM_A_2144614_SM4896.zip › Supplementary files/Suppl Table 2 ROC summary 10 20 2022 Final.pdf]

Supplemental Table 2. ROC analysis summary

Comparison[a]	Cutoff[b]	p-value[c]	AUC[d]	Spec.[e]	Sens.[f]	LHR+[g]	LHR-[h]
**DC vs IE BST**							
Actinomyces odontolyticus	9.4×10^{-4}	<0.0001	0.83	74.1	89.3	6.91	0.29
Granulicatella-bacterium adiacens-enrichment	5.8×10^{-4}	0.024	0.67	90.5	59.3	6.22	0.45
Haemophilus parainfluenzae	<0	<0.0001	0.91	86.9	88.9	6.79	0.13
Lactobacillus rhamnosus	>0	0.0005	0.66	98.8	33.3	28	0.67
Rothia mucilaginosa	4.5×10^{-3}	0.0033	0.71	89.3	59.3	5.53	0.46
Streptococcus mitis	<0	<0.0001	0.87	88.1	88.9	7.47	0.13
Streptococcus parasanguinis	4.2×10^{-3}	<0.0001	0.81	86.9	66.7	5.09	0.38
Streptococcus salivarius	1.0×10^{-3}	<0.0001	0.88	94.1	85.2	14.31	0.16
Streptococcus sanguinis	6.0×10^{-4}	0.0003	0.73	80.9	74.1	3.89	0.32
**DC vs IE sub-g**							
Streptococcus gordonii	1.9×10^{-3}	<0.0001	0.93	81.5	100.0	5.4	0
**DC vs IE sub-g&supra-g**							
Actinomyces viscosus	6.7×10^{-3}	<0.0001	0.63	75.9	87.5	3.63	0.16
Corynebacterium matruchotii	1.9×10^{-3}	<0.0001	0.85	79.6	87.5	4.3	0.16
Fusobacterium nucleatum	4.9×10^{-4}	<0.0001	0.84	87.0	75	5.79	0.29
Lactobacillus rhamnosus	>0	0.0047	0.68	98.2	37.5	20.25	0.64
Rothia dentocariosa	4.2×10^{-3}	0.0279	0.71	79.6	75	3.68	0.31
Streptococcus gordonii	1.9×10^{-3}	<0.0001	0.91	83.3	93.8	5.63	0.075
Streptococcus sanguinis	<0	0.0382	0.70	98.2	50	27	0.51
Veillonella parvula	8.7×10^{-2}	0.0713	0.68	87.0	56.3	4.34	0.5
**HC vs DC BST**							
Haemophilus pittmaniae	<0	0.0018	0.54	8.1	100	1.09	0
NA sp19416	<0	0.0122	0.53	5.4	100	1.06	0
NA sp19816	>0	0.0795	0.52	100.0	3.6		0.96
Peptostreptococcus stomatis	>0	0.0795	0.52	100.0	3.6		0.96
Streptococcus pneumoniae-pseudopneumoniae	<0	0.0005	0.56	13.5	98.8	1.14	0.088
Pseudomonas fluorescens	<0	0.001	0.55	9.0	100.0	1.1	0
Pseudomonas gingeri-putida-syringae	<0	0.0001	0.56	11.7	100.0	1.13	0
Rhizobium radiobacter	<0	0.0065	0.53	6.3	100.0	1.07	0
**HC vs IE BST**							
Actinomyces odontolyticus	1.9×10^{-3}	<0.0001	0.84	88.3	77.8	6.64	0.25
Gemella haemolysans	<0	<0.0001	0.84	80.2	88.9	4.48	0.14
Granulicatella-bacterium adiacens-enrichment	5.8×10^{-4}	0.0202	0.67	91.0	59.3	6.58	0.45
Haemophilus parainfluenzae	6.4×10^{-3}	<0.0001	0.98	89.2	100.0	9.25	0
Lactobacillus fermentum	>0	0.002	0.64	98.2	29.6	16.44	0.72
Lactobacillus rhamnosus	>0	0.0003	0.67	100	33.3		0.67
Rhizobium tropici	>0	0.0009	0.65	100	29.6		0.7
Streptococcus mitis	<0	<0.0001	0.89	91.9	88.9	10.96	0.12
Streptococcus sanguinis	6.0×10^{-4}	0.0001	0.77	8829	74.1	6.32	0.29

Footnote:

[a]Oral sample site data combinations from samples of buccal mucosa (B), saliva (S), tongue (T), supragingival (supra-g), and/or subgingival (sub-g) plaque for patients likely to develop infective endocarditis (IE), non-IE disease controls (DC), and healthy controls (HC).

[b]The Youden's index associated criterion or 'cutoff' value used as the optimum cutoff point for a diagnostic test.

[c]The probability that the observed sample area under the ROC curve is found when the true population is 0.5 (α=0.05).

[d]The area under the curve (AUC).

[e]The probability that a test result will be negative when the species is not present.

[f]The probability that a test result will be positive when the species is present.

[g]Positive likelihood ratio.

[h]Negative likelihood ratio.
