# Supplementary material for: Oral microbiomes of patients with infective endocarditis (IE): a comparative pilot study of IE patients, patients at risk for IE and healthy controls
Source: J Oral Microbiol. 2022 Nov 15;15(1):2144614. doi: 10.1080/20002297.2022.2144614 (PMC9668282; doi:10.1080/20002297.2022.2144614)
Supplement: Supplemental Material [file ZJOM_A_2144614_SM4896.zip › Supplementary files/Suppl Table 3 MIND overlap 10 20 2022 Final.pdf]

Supplemental Table 3. MIND analysis overlap

a. BST sample data combination

**Blood isolate & DC overlap (n=59)**	**Blood isolate & IE overlap (n=7)**
Acinetobacter lwoffii	
Actinomyces naeslundii	*Actinomyces naeslundii*
Aerococcus christensenii	
Anaerococcus prevotii	
Atopobium vaginae	
Bacteroides sp 3_1_33FAA; Bacteroides finegoldii; Bacteroides sp 3_1_40A	
Bifidobacterium breve	
Capnocytophaga ochracea	
Cutibacterium acnes	
Dialister micraerophilus	
	Eikenella corrodens
Finegoldia magna	
Fusobacterium gonidiaformans; Fusobacterium nucleatum	
Gemella haemolysans	
Haemophilus parainfluenzae	*Haemophilus parainfluenzae*
Kocuria rhizophila	
Lactobacillus crispatus; Lactobacillus gasseri; Lactobacillus iners; Lactobacillus jensenii; Lactobacillus vaginalis	
Microbispora rosea	
Micrococcus luteus	*Micrococcus luteus*
Mycoplasma hominis	
Pantoea agglomerans	
Paracoccus aminovorans	
Parvimonas micra	
Porphyromonas asaccharolytica; Porphyromonas bennonis	
Prevotella amnii; Prevotella bivia; Prevotella buccalis; Prevotella disiens; Prevotella intermedia; Prevotella melaninogenica; Prevotella nanceiensis; Prevotella stercorea; Prevotella timonensis	*Prevotella loescheii*
Pseudomonas stutzeri	
Rothia dentocariosa; ***Rothia mucilaginosa***	
Serratia marcescens	
Sneathia amnii; Sneathia sanguinegens	
Staphylococcus epidermidis	
Streptococcus anginosus; Streptococcus gordonii; Streptococcus infantis; Streptococcus intermedius ***Streptococcus mitis****; Streptococcus oralis; Streptococcus pneumoniae*	*Streptococcus gordonii*
Sutterella wadsworthensis	
Veillonella atypica; Veillonella dispar; Veillonella montpellierensis; Veillonella parvula	*Veillonella atypica*

b. sub-g&supra-g sample data combination

**Blood isolate & DC overlap (n=38)**	**Blood isolate & IE overlap (n=27)**
	Acinetobacter lwoffii
Actinomyces naeslundii	*Actinomyces naeslundii*
Aerococcus christensenii	
Anaerococcus prevotii	
Atopobium vaginae	
Bifidobacterium breve	
Capnocytophaga ochracea	*Capnocytophaga ochracea*
Cutibacterium acnes	*Cutibacterium acnes*
Dialister micraerophilus	
	Eikenella corrodens
Finegoldia magna	
Fusobacterium gonidiaformans; ***Fusobacterium nucleatum***	
Gemella haemolysans	
	Haemophilus parainfluenzae
	Janthinobacterium lividum
	Kocuria rhizophila
Lactobacillus crispatus; Lactobacillus gasseri; Lactobacillus iners; Lactobacillus jensenii; Lactobacillus vaginalis	*Lactobacillus iners*
	Microbispora rosea
	Micrococcus luteus
Mycoplasma hominis	
	Pantoea agglomerans
	Paracoccus aminovorans
Parvimonas micra	
Porphyromonas asaccharolytica; Porphyromonas bennonis	
Prevotella amnii; Prevotella bivia; Prevotella buccalis; Prevotella disiens; Prevotella intermedia; Prevotella loescheii; Prevotella melaninogenica; Prevotella timonensis	*Prevotella intermedia; Prevotella loescheii; Prevotella melaninogenica; Prevotella nanceiensis; Prevotella stercorea*
	Pseudomonas stutzeri
	Rothia dentocariosa
	Serratia marcescens
Sneathia amnii; Sneathia sanguinegens	
Staphylococcus epidermidis	
Streptococcus gordonii; *Streptococcus mitis; Streptococcus oralis; Streptococcus sanguinis*	*Streptococcus anginosus; Streptococcus gordonii; Streptococcus infantis*
Sutterella wadsworthensis	
Veillonella atypica; Veillonella montpellierensis	*Veillonella atypica; Veillonella dispar;* ***Veillonella parvula***

Footnote:

Microbial Interaction Network Database (MIND) analysis showing overlapping species interactions for:
a. buccal, saliva, and tongue (BST) sample data combination and,
b. subgingival plaque with supragingival plaque (sub-g&supra-g) sample data combination using LEfSe identified differential species as input for disease controls and Infective Endocarditis (IE) patients compared to identified blood isolates. Input and output species counts were as follows:

Blood isolates: input=7 (*Staphylococcus agalactiae* was not in the database); output=115
BST DC: input=6; output=151
BST IE: input=4 (*Rhizobium tropici* was not found in the database); output=27
sub-g&supra-g DC: input=3 (*Actinomyces viscosus* was not found in the database); output=55
sub-g&supra-g IE: input=5 (*Musa textilis* was not found in the database); output=73

Species shown in bold were used as input and had interactions.
